# Powered ankle-foot orthoses: the effects of the assistance on healthy and impaired users while walking

**DOI:** 10.1186/s12984-018-0424-5

**Published:** 2018-10-01

**Authors:** Marta Moltedo, Tomislav Baček, Tom Verstraten, Carlos Rodriguez-Guerrero, Bram Vanderborght, Dirk Lefeber

**Affiliations:** Department of Mechanical Engineering, R&MM Research Group, and Flanders Make, Vrije Universiteit Brussel (VUB), Pleinlaan 2, Brussels, 1050 Belgium

**Keywords:** Powered ankle-foot orthosis, Robotics, Orthotics, Gait, Wearable robots

## Abstract

In the last two decades, numerous powered ankle-foot orthoses have been developed. Despite similar designs and control strategies being shared by some of these devices, their performance in terms of achieving a comparable goal varies. It has been shown that the effect of powered ankle-foot orthoses on healthy users is altered by some factors of the testing protocol. This paper provides an overview of the effect of powered walking on healthy and weakened users. It identifies a set of key factors influencing the performance of powered ankle-foot orthoses, and it presents the effects of these factors on healthy subjects, highlighting the similarities and differences of the results obtained in different works. Furthermore, the outcomes of studies performed on elderly and impaired subjects walking with powered ankle-foot orthoses are compared, to outline the effects of powered walking on these users. This article shows that several factors mutually influence the performance of powered ankle-foot orthoses on their users and, for this reason, the determination of their effects on the user is not straightforward. One of the key factors is the adaptation of users to provided assistance. This factor is very important for the assessment of the effects of powered ankle-foot orthoses on users, however, it is not always reported by studies. Moreover, future works should report, together with the results, the list of influencing factors used in the protocol, to facilitate the comparison of the obtained results. This article also underlines the need for a standardized method to benchmark the actuators of powered ankle-foot orthoses, which would ease the comparison of results between the performed studies. In this paper, the lack of studies on elderly and impaired subjects is highlighted. The insufficiency of these studies makes it difficult to assess the effects of powered ankle-foot orthoses on these users.

To summarize, this article provides a detailed overview of the work performed on powered ankle-foot orthoses, presenting and analyzing the results obtained, but also emphasizing topics on which more research is still required.

## Background

**Fig. 1:**
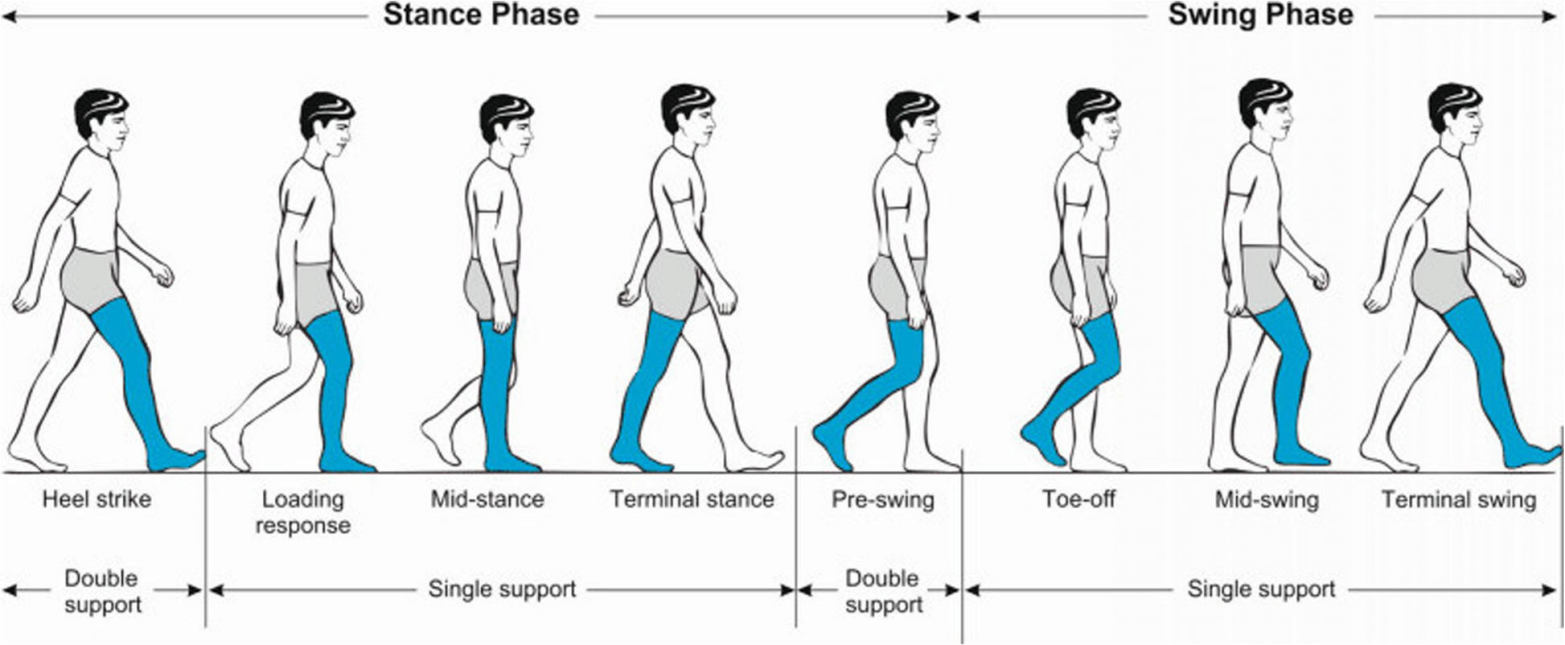
Illustration of the gait cycle. After heel strike, during the loading response, the ankle plantarflexes in a controlled manner to allow the foot to make a gradual contact with the ground. During mid-stance, the ankle dorsiflexes while the stance leg moves the bodyweight over the forefoot. In the terminal stance, a propulsive force is generated by the ankle plantarflexors to initiate leg swing, generate forward velocity and redirect the body’s center of mass [78, 79]; this phase of the gait cycle is also called push-off. After the toe-off, during swing phase, the ankle dorsiflexes to allow toe clearance and prepare the foot for the next heel strike. Picture is taken from [80]

Walking is the most common form of locomotion to move from one place to another. Despite its apparent simplicity, it is a complex movement that requires a precise coordination of multiple body segments and muscles. Although the human gait pattern appears to be energetically optimized [1], walking still requires a large amount of metabolic energy. One of the major determinants of this energetic cost is the mechanical work performed at the ankle joint to redirect the center of mass during step-to-step transitions [1–3]. The human ankle has distinctive functions in each phase of the gait cycle [4, 5], as shown in Fig. 1.

The capability of the ankle joint to deliver these functionalities can be reduced as a consequence of aging, pathologies, and injuries. It has been shown that the elderly walk slower, take shorter steps, and exhibit a smaller range of motion in the joints of the lower limbs [6, 7]. Furthermore, aging causes larger deficits in torque production in the ankle plantarflexors as compared to other muscle groups. For this reason, to maintain the same walking speed as young subjects, the elderly redistribute the contribution of the torques and powers provided by the lower limbs joints, thus, increasing the effort at the hip extensors and decreasing it at the knee extensors and plantarflexors with respect to young subjects [7].

Subjects with weakened capabilities of the ankle joint due to injuries or diseases, such as strokes, hemiplegia, and incomplete spinal cord injuries, show an altered gait pattern when compared to that of healthy subjects [8–10]. The outcomes of ankle muscles deficiencies can be divided into two categories, depending on the muscle group involved. Weakened dorsiflexors result in a steppage gait pattern, commonly called drop foot. The main consequences of drop foot are foot slap during the loading response and toe drag during swing. In subjects with drop foot, the reduced foot clearance during the swing is often compensated by a pelvic hike, circumduction, or vaulting [10, 11]. On the other hand, weakened ankle plantarflexors reduce the torque provided at push-off and affect the subject’s stability during single support, which is counteracted by a shortening of the contralateral step length. In most cases, the reduced propulsive torque causes a reduction of the subject’s walking speed. In some cases, however, the subject maintains a faster walking speed by compensating for the weakened plantarflexor muscles with the hip flexors [12]. However, this compensation can negatively affect the metabolic cost of walking [13]. Compared to healthy subjects, whose gait pattern is characterized by symmetry between the left and right spatial and temporal parameters [4], patients affected by unilateral deficits, as is the case with hemiplegic patients, present a significant asymmetry in the gait characteristics between the sound and the affected lower limb [10, 14–16].

The crucial role of the ankle joint in human walking, in the last two decades, led to the development of numerous powered ankle-foot orthoses (PAFOs). Their aim is to improve the gait pattern of impaired users or decrease the biological effort of healthy subjects during walking. Some of the developed PAFOs share the same combination of type of actuators and type of controllers [17], but their performance differs with different studies. Recently, researchers have started analyzing the influence of certain parameters of the control strategy on the performance of the assistance provided by the PAFO to healthy subjects [18–21]. However, an extensive comparison of the results found in different works, performed on both healthy and weakened subjects walking with PAFOs, is missing.

The aim of this paper is to collect the results of studies assessing the assistance provided by PAFOs while walking on healthy and impaired users and compare their outcomes to give an overview of the effects of walking with a PAFO in both groups of subjects. For this purpose, in this paper only articles which analyzed the effects of PAFOs on users during walking experiments were included. On the other hand, articles that reported exclusively on the design of PAFOs, the results of characterization tests which did not involve users, or in which the protocol of the experiments did not involve walking trials, were excluded. Furthermore, articles involving walking experiments in which the discussed results were only about the performance of the actuator of the PAFO and not its effect on the user, were also omitted. Some studies that were performed with a soft exosuit providing ankle assistance on healthy and impaired users were included, due to the relevance of their findings with respect to the aspects discussed by this article. However, it is important to highlight that these devices also provide passive hip assistance along with active ankle assistance [22–27]. The rest of the paper is structured as follows: first, an overview of the developed PAFOs is given; then, the effects of the assistance provided by PAFOs on healthy and weakened users are compared and discussed. Concluding remarks on the presented results are given at the end of the paper.

## PAFOs classification

As mentioned above, numerous PAFOs have been developed recently to assist healthy and impaired users while walking. With respect to their main goal, they can be divided into four distinctive groups as follows [28]:

- **Basic Science PAFOs**: PAFOs that have been developed to study human physiology and biomechanics by analyzing the user’s response to external ankle actuation (Table 1);
- **Augmentation PAFOs**: PAFOs whose goal is to increase the walking endurance of healthy users, by reducing their metabolic cost and/or muscle effort (Table 2);
- **Assistive PAFOs**: PAFOs that aim to assist users with impaired ankle capabilities to bring their performance closer to that of healthy individuals (Table 3);
- **Rehabilitation PAFOs**: PAFOs whose goal is to rehabilitate subjects who suffered an injury or illness and to re-train their walking capabilities to pre-injury ones (Table 4).

**Table 1:** Basic science PAFOs tested on healthy subjects in walking experiments

Ref.	Actuation (Pf/Df)	Control	Weight PAFO	Subjects	Conditions (n. sessions / repetitions)	Ul / Bl	Portable	Aim	Outcomes
Ferris, 2005 [29]	1PAM (Pf) + 1PAM (Df)	PMc (SOL / TIB)	1.6 kg	1 H	training: 60 min; test: 6N-6U-30P(Pf)-30P(Df) (1sess)	/	no	Design lightweight orthosis for powered Pf and Df	Pf assist: SOL reduced 47%, TIB increased 10% w.r.t. U; Df assist: SOL increased 10%, TIB reduced 20% w.r.t. U
Ferris, 2006 [30]	1PAM (Pf) + 1PAM (Df)	PMc (SOL / TIB)	1.7 kg	1 H	P (1 sess)	/	no	Improve design of PAFO in Ferris, 2005 [29]	Design improved: easier to don-doff, more comfortable
Galle, 2013 [43]	PAM (Pf)	P-Bc	0.76 kg	9 H	24P-4U	Bl	no	Assess the effects of adaptation on the users	Metabolic cost of walking and lower limbs muscle activation decrease from beginning to adapted period
Gordon, 2006 [44]	1 / 2 PAM (Pf)	P-Bc	1.3 / 1.7 kg	3 H	training: 1 min, test: 20secN-20secU-20secP (2 muscle configurations and 4 speeds) (2sess)	Ul	no	Examine effect of walking speed and amount of assistance	Ankle more plantarflexed in double PAM configuration, but similar total moments
				1 H	training: 1 min; test: 20secU-20secP (3 muscle lengths)		no	Examine the effect of PAM length	Middle length of PAM is optimal
Gordon, 2007 [31]	PAM (Pf)	PMc (SOL)	1.2 kg	10 H	10U-30P-15U (2 sess)	Ul	no	Examine adaptation of users and whether adapted pattern is retained in time	Users learn to use less their muscles when assistance is provided and can retain this information
Kao, 2009 [32]	PAM (Df)	PMc (TIB) continuous	/	5 H	10U-30P-15U (2 sess)	Ul	no	Examine how subjects adapt to Df assistance	TIB reduced only in initial stance in group continuous; in both groups TIB activity in swing is similar to U
		PMc (TIB) only in swing		5 H					
Kao, 2010 [33]	2 PAM (Pf)	PMc (SOL)	1.08 kg	11 H	10U-30P-15U (2 sess)	Ul	no	Determine if adaptation depends on the amount of assistance provided	Higher amount of assistance leads to longer adaptation time
Kinnaird, 2009 [34]	PAM (Pf)	PMc (MG)	1.23 kg	10 H	10U-30P-15U (2 sess)	Ul	no	Assess how nervous system adapts to external assistance	Main reduction in SOL (muscle replaced by actuation), however also MG reduced to modulate action PAFO

The type of actuator, the weight and portability of the device, the protocol, the main goal and outcomes of each study are reported

*H*: healthy users; *PAM*: pneumatic artificial muscle; *P*-*Bc*, *PMc*: phase-based and proportional myoelectric controller; *Pf*, *Df*: plantarflexion and dorsiflexion; *SOL*, *TIB*, *MG*: soleus, tibialis anterior and medial gastrocnemius muscles; *N*, *U*, *P*: normal walking, unpowered and powered walking condition; *Ul*, *Bl*: uni-/bi-lateral PAFO; the protocol code defines the conditions and the timings in minutes (unless contrary indication) used for each session, e.g. xPyUzN means x minutes of powered walking, y minutes of unpowered walking and z minutes of normal walking. In the works in which different experiments were performed (for example, different control strategies) the common information between the experiments (for example, same condition) is reported only once

**Table 2:** Augmentation PAFOs tested on healthy subjects in level or uphill walking experiments

Ref.	Actuation (Pf/Df)	Control	Weight PAFO	Subjects	Conditions (n. sessions /repetitions)	Ul / Bl	Portable	Results on metabolic cost	Results on muscle activation
Asbeck, 2015 [22]	SEA (Pf*)	P-Bc	10.1 kg	5 H	training:10U-10P; test:8U-8P(x6 powered conditions, different peak passive and active moments)-8U, 34.6kg load (1sess)	Bl	yes	only in 1 condition reduced w.r.t. U (-6.4%)	/
Cain, 2007 [35]	PAM (Pf)	P-Bc	1.1 kg	6 H	N (1sess), 10U-30P-15U (2sess)	Ul	no	/	SOL, LG, MG similar to U
		PMc (SOL)		6 H					SOL, LG, MG reduced wrt U
Galle, 2014 [45]	PAM (Pf)	P-Bc	0.76 kg	9 H	U and P, 15% In, every 3 min load = 5% bodyweight added until exhaustion (1sess)	Bl	no	-10% in P and U at exhaustion, but longer walking time in P w.r.t. U	/
Galle, 2015 [19]	PAM (Pf)	P-Bc	0.76 kg	7 H	training: 24P 0% In; test: 15% In, 4U, 4P(x4 poweredconditions, different onset timings) (1sess)	Bl	no	bigger reduction (-12% w.r.t. U) when onset at 26% and 34%	for onset 34%: TIB increased beginning swing, VL and BIC reduced beginning stride
Galle, 2017 [21]	PAM (Pf)	P-Bc	0.89 kg	14 H	training: 4N-4U-4P(x12) (1sess); tests: 2N-2U-2P(x12) (1sess); 12 powered conditions: 4 onset timings, 3 power levels	Bl	no	bigger reduction for 43% onset and middle power condition (- 21% w.r.t. U)	SOL: reduced with higher power and earlier timings; MG: reduced with higher power and later timings; TIB: increased with increase power
Jackson, 2015 [20]	SEA (Pf)	P-Bc	0.83 kg	8 H	6N-8U-8P(x7 powered conditions: 4 work conditions, 3 torque conditions) (2 sess)	Ul	no	decreased with increased net work, but increased with increasing average torque	Exo-side SOL decreased with increased torque and work; contralateral VL decreased with increased work
Koller, 2015 [40]	PAM (Pf)	Ag-PMc (SOL)	2.08 kg	8 H	10U-30P-10U (3 sess)	Bl	no	reduced throughout sessions; 3rd sess: -18% w.r.t U	1st sess: SOL -20%, RFEM -9%, BIC -18%; 3rd sess: SOL -11%, RFEM -20%, BIC -17% w.r.t. U
Koller, 2017 [41]	PAM (Pf)	Ag-PMc (SOL)	/	8 H	training: 10U-30P-10U (3 sess); test: 10U-10P(Ag-PMc)-10P(P-Bc)-10P(Ag-PMc) (1sess)	Bl	no	similar reduction w.r.t. U with both controllers (-19%)	SOL: reduced 12% more in P-Bc than Ag-PMc w.r.t. U
		P-Bc							
Koller, 2018 [42]	PAM (Pf)	Ag-PMc (SOL)	2.08kg	8 H	training: 10U-30P-10U (3 sess); test: 10U-10P(Ag-PMc)-10P(P-Bc)-10P(Ag-PMc) (1sess)	Bl	no	similar reduction w.r.t. U with both controllers (-19%)	SOL: reduced 19% (peak linear envelope reduced 29%) w.r.t. U; RFEM: reduced 13% (peak linear envelope reduced 39%) w.r.t. U
		P-Bc							SOL: reduced 28% (peak linear envelope reduced 38%) w.r.t. U, SOL activity in P-Bc 11% lower than in Ag-PMc; RFEM: reduced 9% (peak linear envelope reduced 35%) w.r.t. U
Lee, 2016 [23]	SEA (Pf*)	P-Bc	0.89 kg	7 H	8U-8P (x3 powered conditions, different power levels), 23kg load (1 sess)	Bl	no	-(11%-15%) w.r.t. U	/
Malcolm, 2013 [18]	PAM (Pf)	P-Bc	0.67 kg	8 H	4U-4P(x5 powered conditions, different onset timings) (1sess)	Bl	no	bigger reduction (-17% w.r.t. U) when onset at 43%	
Malcolm, 2017 [25]	SEA (Pf*)	P-Bc	1 kg	8 H	training: 8P (x4 powered conditions, different power levels) (1sess); test: 8U-8P (x4 powered conditions, different power levels) (1 sess), 23kg load	Bl	no	-(11%-15%) w.r.t. U	/
Mooney, 2014 [47]	SEA (Pf)	P-Bc	4 kg	7 H	N-P, 23kg load (1 sess)	Bl	yes	reduced w.r.t. N	/
Mooney, 2014 [48]	SEA (Pf)	P-Bc	3.6 kg	7 H	10N-20P-20U-10N (1sess)	Bl	yes	-14% w.r.t. N, U	/
Mooney, 2016 [49]	SEA (Pf)	P-Bc	3.6 kg	6 H	P-U-N (1sess)	Bl	yes	-14% w.r.t. N, U	/
Quinlivan, 2017 [24]	SEA (Pf*)	P-Bc	0.89 kg	7 H	training: 8warm-up-5U(x2)-5P(x4 powered conditions, different peak moments) (1sess); test: 8warm-up-5U(x2)-5P(x4 powered conditions, different peak moments) (1sess);	Bl	no	decreased with increased peak ankle moment w.r.t. U	/
Sawicki, 2008 [36]	PAM (Pf)	PMc (SOL)	1.21 kg	9 H	10U-30P-15U (3 sess)	Bl	no	reduced throughout sessions; 3rd sess: -10% w.r.t U	SOL: reduced throughout sessions; 3rd sess: SOL -28%, MG: -10%, LG: -4% w.r.t. U
Sawicki, 2009 [37]	PAM (Pf)	PMc (SOL)	1.18 kg	10 H	>90 min training; 7U-7P (4 walking speeds) (1sess)	Bl	no	-(10%-12%) w.r.t. U for every walking speed	SOL, MG, LG, TIB: reduced at higher speeds, no difference at lower speeds
Sawicki, 2009 [38]	PAM (Pf)	PMc (SOL)	1.18 kg	9 H	>90 min training; 7U-7P (0%, 5%,10%, 15% In) (1sess)	Bl	no	-(10%-13%) w.r.t. U for every incline	SOL: -25% at 0% In, -(16%-18%) with In; LG: -24% in 0% In, -(8%-15%) with In
VanDijk, 2017 [50]	SEA (Pf)	P-Bc	9 kg	7 H	12U-12P-12N (1sess)	Bl	yes	increased w.r.t. U	/
Zhang, 2017 [46]	SEA (Pf)	P-Bc	0.83 kg	1 - 11 H	N-U-64P, several conditions	Ul and Bl	no	optimized pattern changes with subjects; higher metabolic cost reduction with optimized assistance w.r.t. generalized	SOL: -36% w.r.t N, -41% w.r.t. U

For uphill walking, the inclination is indicated. The type of actuator, the weight and portability of the device, the protocol and the results regarding the reduction of metabolic cost or muscle activation of each study are reported

H: healthy users; In: inclination; PAM: Pneumatic artificial muscle; SEA: Series elastic actuator; Pf: Plantarflexion; P-Bc: Phase-based controller; PMc, Ag-PMc: Proportional myoelectric controller and adaptive gain PMc; SOL, LG, MG, TIB, RFEM, BIC: VL: soleus, lateral and medial gastrocnemius, tibialis anterior, rectus femoris, biceps femoris and vastus lateralis muscles; N, U, P: normal walking, unpowered and powered walking condition; Ul, Bl: uni-/bi-lateral PAFO; the protocol code defines the conditions and the timings in minutes used for each session, e.g. xPyUzN means x minutes of powered walking, y minutes of unpowered walking and z minutes of normal walking. *: the Pf module exerted also hip flexion torques. In the works in which different experiments were performed (for example, different control strategies) the common information between the experiments (for example, same condition) is reported only once

**Table 3:** Assistive PAFOs tested on impaired and elderly subjects

Ref.	Actuation (Pf/Df)	Control	Weight PAFO	Subjects	Conditions (n. sessions / repetitions)	Walking speed	Ul / Bl	Portable	Outcomes
Awad, 2017 [63]	SEA (Pf + Df)	P-Bc: Pf assist during stance, Df assist during swing	0.9 kg	8 Str	8P-8U	Self-selected	Ul	no	*↓* hip hiking *↓* circumduction *↑* step length *↑* Df in swing
Awad, 2017 [64]	SEA (Pf + Df)	P-Bc: Pf assist during stance, Df assist during swing	0.9 kg	9 Str	8P-8U (2sess, 2 different onset timings)	Self-selected	Ul	no	*↑* Df in swing *↓* propulsion asymmetry *↓* metabolic cost
			4.1 kg		P-U			yes	*↑* Df in swing *↓* propulsion asymmetry
Bae, 2015 [26]	SEA (Pf* + Df)	P-Bc: Pf assist during PO, Df assist during swing	0.9 kg	3 Str	baseline: N; training: P (3 to 5 sess); test: P	Self-selected	Ul	no	*↑* step and stance time symmetry *↑* propulsion symmetry *↓* circumduction
Bae, 2018 [27]	SEA (Pf* + Df)	P-Bc: Pf assist during stance, Df assist during swing	0.9 kg	7 Str	8P-8U	Self-selected	Ul	no	*↑* symmetry body CoM power generation *↓* metabolic cost *↑* symmetry ankle power generation
Blaya, 2004 [51]	SEA (Df)	P-Bc: Df assist during LR and swing	2.6 kg	3 H	N-A-P	Self-selected (1.21m/s (H), 1.15m/s (Dfi)), slow, fast (decreased, increased 25%)	/	no	/
				2 Dfi					*↑* Df in swing; *↑* Pf in stance; *↓* occurrence of foot slap at all speeds; *↓* step length and step time asymmetry at slow and self-selected speed
Galle, 2017 [56]	PAM (Pf)	P-Bc: Pf assist during PO	0.76 kg	8 E	5N-5U-5P(x2 powered conditions) (2 sess)	1.11 m/s	Bl	no	*↑* step length *↓* metabolic cost
Norris, 2007 [55]	PAM (Pf)	P-Bc: Pf assist during PO	0.8 kg	9 H	N-U-P	Self-selected	Bl	no	*↑* walking speed; modest reduction metabolic cost
				7 E					no difference in walking speed; modest reduction metabolic cost
Sawicki, 2006 [65]	PAM (Pf) + elastic cord (Df)	P-btnc: PO assist	1.09 kg	5 iSCI	10/15N-10/15U-10/15P(therapist)-10/15P(patient), 30/50% BWS (2 sess)	0.36, 0.54, 0.72, 0.89 m/s (subjects preferred: 0.56 m/s)	Bl	no	*↑* RoM; *↑* Pf in stance; bigger improvements in therapist than patients control; in P muscle activity similar to N
Shorter, 2011 [52]	BPnA (Pf + Df)	P-Bc: Df assist during LR and swing, Pf assist during PO	3.1 kg	3 H	1N-1U-1P(x3 powered conditions)	Self-selected	Ul	yes	*↓* tibialis anterior activity
				1 Pfi	1.5N-1.5U-1.5P(x3 powered conditions)		Ul + AFO		improved Pf; improved PO phase
Shorter, 2011 [53]	BPnA (Pf + Df)	P-Bc: Pf assist during PO	3.1 kg	1 Pfi	N-U-P	Self-selected	Ul + AFO	yes	*↓* RoM in Df; *↓* symmetry; improved PO phase
		P-Bc: Df assist during LR and swing		1 Dfi					*↓* occurrence foot drop; better foot positioning heel strike; *↓* symmetry
Takahashi, 2015 [39]	PAM (Pf)	PMc (impaired soleus & GRF)	/	5 Str	5N-5U-5P (x3 powered conditions)	75% of self-selected	Ul	no	*↓* impaired soleus activation; trend of decreasing metabolic cost
Yeung, 2017 [54]	StA (Pf + Df)	P-Bc: Pf assist during LR and PO, Df assist during swing	1 kg	3 Str	training: 10U; test: N-U-P	/	/	yes	*↓* occurrence foot drop; no enhancement PO

The type of actuator, the weight and portability of the device, the protocol and the main outcomes of each study are reported. In some studies the devices were tested also on healthy young users

iSCI, Str, Dfi/Pfi: Incomplete spinal cord injury, stroke and dorsi-/plantar-flexion impaired subjects; E: Elderly subjects; H: Healthy young subjects; SEA: Series elastic actuator; PAM: Pneumatic artificial muscle; BPnA: Bidirectional rotaty pneumatic actuator; StA: Stiff actuator; Pf, Df: Plantarflexion and dorsiflexion; P-Bc: Phase-based controller; P-btnc: push-button controller, PMc: Proportional myoelectric controller; LR: Loading response; PO: Push-off; N, A, U, P: Normal walking, walking with conventional AFO, unpowered and powered walking condition; BWS: Bodyweight support; GRF: Ground reaction force; Ul, Bl: Uni-/bi-lateral PAFO; CoM: Center of mass; the protocol code defines the conditions and the timings in minutes used for each session, e.g. xPyUzN means x minutes of powered walking, y minutes of unpowered walking and z minutes of normal walking. *: the Pf module exerted also hip flexion torques. In the works in which different experiments were performed (for example, different control strategies) the common information between the experiments (for example, same condition) is reported only once

**Table 4:** Rehabilitation PAFOs tested on hemiplegic and stroke patient

Ref.	Actuation (Pf/Df)	Control	weight PAFO	Subjects	Training and comparisons	Walking Speed	Ul / Bl	Portable	Outcomes
Bharadwaj, 2005 [61]	SOM (Pf + Df), passive inv + ev	P-Bc	/	1 H	/	fixed gait time	Ul	/	lower RoM than in N
Hwang, 2006 [57]	SEA (Pf + Df)	P-Bc: Pf assist during LR and PO, Df assist during MSt and swing	2.8 kg	5 H	N-A-P	/	Ul	no	AFO leads to inefficient walking; in P *↑* PO torque
Kim, 2007 [59]	SEA (Pf + Df)	P-Bc: Pf assist during LR and PO, Df assist during MSt and swing	2.8 kg	1 Hem	Training: 4 weeks; Test: 30 min + N-A-P	/	Ul	no	*↑* walking speed and cadence; *↓* asymmetry; prevented foot drop; *↑* Df RoM; improved Pf in PO
Kim, 2011 [58]	SEA (Pf + Df)	P-Bc: Pf assist during LR and PO, Df assist during MSt and swing	2.8 kg	3 Hem	Training: 4 weeks; Test: 30 min + N-A-P	/	Ul	no	*↑* walking speed and cadence; *↓* asymmetry; prevented foot drop; *↑* Df RoM; improved Pf in PO
Ward, 2007 [62]	SOM (Pf + Df), passive inv + ev	P-Bc	/	1 Str	Training: 8 weeks; Pre-, mid- and post-tests: N	Self-selected	Ul	/	improved kinematics; *↑* walking cadence; for some tests results in line with training without PAFO
Ward, 2011 [60]	SEA (Pf)	P-Bc: assist PO	/	3 Str	Training: 3 weeks; Pre- and post-test: N	Self-selected	Ul	no	*↑* cadence; *↑* RoM

The type of actuator, the weight and portability of the device, the training and testing sessions and the main outcomes of each study are reported. In some studies, the devices were tested also on healthy users

Hem, Str: Hemiplegic and stroke patients; H: Healthy users; SEA: Series elastic actuator; SOM: Double-acting spring over muscle actuator; Pf, Df: Plantarflexion and dorsiflexion; inv, ev: Inversion and eversion; P-Bc: Phase-based controller; LR: Loading response; PO: Push-off; MSt: mid-stance; N, A, P: Normal walking, walking with conventional AFO, PAFO powered walking; Ul, Bl: Uni-/bi-lateral PAFO

Tables 1, 2, 3 and 4 summarize the main outcomes of studies on walking experiments performed on the four groups of PAFOs. Within the same group, different actuation combinations, such as applied controllers, actuation principles and directions of the assistance, and designs have been developed. The categorization of different devices in terms of these aspects can be found in the sequel.

*Type of controllers*

 The controllers used in the different PAFOs can be divided into the following categories:

- **Proportional Myoelectric Controller (PMc)**, in which the action of the PAFO is proportional to the activity of a predefined user’s muscle [29–39];
- **Adaptive Gain Proportional Myoelectric Controller (Ag-PMc)**, in which the proportional gain of the PMc is adjusted to have the maximal peak actuation with each stride, even when the user’s maximal muscle activity differs between strides [40–42];
- **Phase-Based Controller (P-Bc)**, in which the actuation of the PAFO is based on the detection of certain gait events [18–27, 35, 41–64];
- **Push-Button Controller (P-btnc)**, in which the actuation is proportional to the displacement of a push-button [65].

The P-Bc and PMc are the most common types of controllers used in basic science (Table 1) and augmentation (Table 2) PAFOs. Assistive (Table 3) and rehabilitation (Table 4) PAFOs are mainly controlled by a P-Bc.

*The actuation principles*

The different types of actuators used in the PAFOs presented in Tables 1, 2, 3 and 4 are as follows:

- **SEA**: series elastic actuator [66], which consists of an electric motor in series with an element with spring-like behavior [20, 22–27, 46–51, 57–60, 63, 64];
- **PnA**: pneumatic actuators, which transform energy from pressurized air into motion. The most common type of PnA used in PAFOs is the PAM (pneumatic artificial muscle) [67], which is a contractile device that expands and shortens by means of pressurized air [18, 19, 21, 29–45, 55, 56, 65]. When the PAM is attached in parallel with a standard compression spring, the actuator is called a double-acting spring over muscle actuator (**SOM**) [61, 62]. Another subcategory of the PnA is the bidirectional rotary pneumatic actuator (BPnA), presented in [52, 53];
- **StA**: stiff actuators, which are actuators that do not have intended spring behavior [54].

PnA is the most common actuation principle used in basic science, augmentation, and assistive PAFOs (Tables 1, 2 and 3), while the majority of the rehabilitation PAFOs use SEAs (Table 4).

*The direction of actuation*

The great majority of developed PAFOs are unidirectional devices providing only plantarflexion assistance [18–21, 31, 33–50, 55, 56, 60]. This is the case of augmentation PAFOs, whose goal is to reduce the biological effort of the user by providing powered plantarflexion during push-off [18–21, 35–38, 40–42, 45–50]. The works presented in [22–25] can also be grouped in the category of augmentation PAFOs providing only powered plantarflexion during push-off; however, it is important to highlight that in these studies, a hip flexion moment is provided together with the plantarflexion moment, due to the textile architecture of the exosuit. Plantarflexion assistance is provided by assistive or rehabilitation PAFOs to improve the gait pattern of impaired subjects, increase their walking speed, and enhance their propulsion [39, 55, 56, 60]. A smaller group of PAFOs provides only dorsiflexion assistance to the user [32, 51], which is of great importance for drop foot patients. Some of the developed PAFOs combine the assistance in both directions of the sagittal plane for assistive [52–54, 63–65] and rehabilitative [57–59, 61, 62] purposes, or to study the effects of powered walking on users [29, 30]. In the works presented in [26, 27], impaired users are assisted in both plantarflexion and dorsiflexion movements while walking; however, in these works, a hip flexion moment is provided together with the plantarflexion moment, due to the textile architecture of the exosuit. Between the PAFOs presented in Tables 1, 2, 3 and 4, only the one tested in [61, 62] implements an inversion/eversion degree of freedom, however, the PAFO was controlled only in the sagittal plane during the tests.

*Portable and tethered designs*

From Tables 1, 2, 3 and 4, it is possible to observe that only a few of the presented PAFOs are portable devices [22, 47–50, 52–54, 61, 64]. Tethered devices are suitable for cases in which the aim of the PAFO is related to studies of human physiology performed in laboratories or the rehabilitation of impaired subjects in hospitals or rehabilitation centers. When the goal of the PAFO is to assist impaired users in their daily life activities or augment the walking capabilities of healthy users, the portability of the device is a key requirement. However, the design of an efficient, but lightweight PAFO is still a challenge [68].

The advantage of tethered PAFOs is the possibility of placing heavy components off-board, leading to more lightweight devices with a lower negative impact of the PAFO on the user. In the design of PAFOs, the distribution of added weight on the lower limbs has to be carefully considered. Mass added at the user’s lower limbs increases the moment of inertia of the legs, which increments the metabolic cost of walking [69]. Furthermore, the metabolic cost of walking increases with a more distal location of the added mass. For this reason, foot loads are considerably more expensive than the loads placed in other locations [69]. To be comfortably worn, the maximum weights added on the user’s segments should not exceed 15% and 1.25% of the user’s body weight when placed on the torso and each foot, respectively [70]. Thus, considering a subject of 75 kg, the weight of a device placed on his/her foot should not exceed 0.94 kg, while on the torso he/she can carry 11.25 kg. The weight of the PAFO is, thus, a critical factor, especially when the objective is the reduction of a user’s effort. This explains why most of augmentation PAFOs are tethered devices. Few untethered augmentation PAFOs exist [22, 47–50], and they are used to assess the possibility of reducing the metabolic cost of walking, at the cost of a higher weight that needs to be compensated by the assistance provided by the PAFO.

## Factors influencing the performance of augmentation PAFOs

The aim of augmentation PAFOs is to enhance the walking capabilities of healthy young users by providing powered push-off (Table 2). There are two main goals of these PAFOs: the reduction of the user’s metabolic cost of walking and the reduction of the lower limb muscle effort. These effects are indicators that the PAFO is able to partly replace the function of a user’s biological ankle or to augment it in favor of a higher metabolic benefit.

The performance of augmentation PAFOs in reducing the biological effort of users depends on several factors:

- the adaptation of the user to powered assistance;
- the timing of the actuation profile;
- the assistance magnitude, defined as the average power provided by the PAFO during one stride at one leg of the user;
- the type of controller.

The last three factors in the list are parameters defined in the protocol and they are called in this paper assistance parameters.

In this section, the effects of these factors on the reduction of the metabolic cost of walking and the muscle effort of the user are individually analyzed.

### Adaptation of healthy subjects to the assistance

It is known that subjects need a certain period, called the adaptation period, to get used to the external assistance provided by the PAFO. In this period, they learn how to take advantage of powered assistance and to optimize their walking pattern in the powered condition. At the beginning of powered walking, when the subject is not yet adapted, his/her kinematics, muscle activation and metabolic cost are altered with respect to unpowered walking [18, 31–36, 40, 43]. During the adaptation period, the subjects reach a steady state. In this state, the ankle kinematics and muscle activation patterns return to a similar state as they were during normal walking, despite some deviations. The most common deviations are the reduced amplitude of the activity of the muscles working in unison with the actuation [31–36, 40, 43] and some differences in the ankle kinematics, which are more plantarflexed when walking with a powered plantarflexion [33, 36, 40, 43] or more dorsiflexed when walking with a powered dorsiflexion [32]. However, other deviations such as the increase in the muscle activity of lower limb muscles have been found during the adapted period, with respect to unpowered walking [43]. Furthermore, the metabolic cost of walking reaches a steady-state value, which is, in some cases, lower than during the unpowered condition [18, 36, 40, 43].

Table 5 collects the results reported from studies assessing the adaptation time of different parameters in healthy subjects. The outcomes of these studies are discussed below.

**Table 5:** Comparison of adaptation time in different studies

Ref.	Control	Ul / Bl	Onset	Peak	Parameter	Adaptation
			timing	torque		time
						(*n*^*th*^ session)
Cain, 2007	PMc	Ul	25%	0.6 Nm/kg	kinematics	28 min (1)
[35] △	(SOL)					7 min (2)
					SOL	18 min (1)
						5 min (2)
	P-Bc	UI	25%	0.6 Nm/kg	kinematics	28 min (1)
						7 min (2)
					SOL	18 min (1)
						4 min (2)
Galle, 2013	P-Bc	BI	43%	/	kinematics	4 min (1)
[43] ♢					metab. cost	19 min (1)
Gordon, 2007	PMc	UI	27%	0.5 Nm/kg	kinematics	24 min (1)
[31] △	(SOL)					6 min (2)
					SOL	24 min (1)
						6 min (2)
Kao, 2010	PMc	Ul	15%	0.7 Nm/kg	SOL	>30 min (1)
[33] △	(SOL)					>30 min (2)
Kinnaird, 2009	PMc	UI	/	0.6 Nm/kg	kinematics	25 min (1)
[34] △	(MG)					6 min (2)
					MG	22 min (1)
						5 min (2)
					SOL	19 min (1)
						4 min (2)
Koller, 2015	Ag-PMc	Bl	11%	0.6 Nm/kg	metab. cost	<30 min (1)
[40] △	(SOL)					<30 min (2)
						<30 min (3)
Koller, 2017	Ag-PMc	BI	/	/	metab. cost	<90 min
[41] △	(SOL)					(30 min x (3))
Sawicki, 2008	PMc	Bl	/	0.5 Nm/kg	metab. cost	∼90 min
[36] △	(SOL)					(30 min x (3))

The adaptation time for ankle kinematics, muscle activity, and metabolic cost of walking for healthy users with respect to the onset timing (reported as a percentage of the gait time) and the peak torque provided by the PAFO. The works presented by Sawicki et al. [36] and Koller et al. [40, 41] are included, but they reported on whether the subjects reached a steady state without measuring the exact adaptation time

PMc, Ag-PMc: Proportional myoelectric controller and adaptive gain PMc, P-Bc: Phase-based controller; SOL, MG: Soleus and medial gastrocnemius muscle; Ul, Bl: Uni-/bi-lateral PAFO. Symbols (△,♢) indicate studies performed by the same research group on similar actuation setups. Symbols are consistent between tables. In the works in which different experiments were performed (for example, different parameters) the common information between the experiments (for example, same control strategy) is reported only once

#### Shortening of the adaptation time with multiple sessions

In the studies where several identical sessions were held on different days [31, 33–36, 40], the subjects reached a steady state increasingly faster, over different sessions. This result shows that the subjects could retain the walking pattern used in previous powered trials, however, the different sessions were separated by only two to five days. None of the studies assessed whether this information is erased after a longer period of walking without a powered PAFO and, if that is the case, for how long it can be retained by the subjects.

Table 5 shows that the amount of time to reach a steady state changes with different parameters. Looking at the reported results, there seems to be a trend of ankle kinematics reaching a steady state in a longer period with respect to muscle activation. Furthermore, this effect seems to be independent of the type of controller used [35].

#### Does the testing protocol influence the adaptation time?

Some of the studies reported in Table 5 stand out for their different outcomes.

Galle et al. [43] reported an adaptation time of the ankle kinematics which was much shorter in a single powered session with respect to other studies. Two differences between the study by Galle et al. [43] and the other works in Table 5 assessing the adaptation time in ankle kinematics are the use of a later onset timing and the bilateral, instead of unilateral, use of the PAFO. However, as highlighted in Table 5, this study was performed with a different actuation setup as compared to the other studies, reported in the table. For this reason, it is difficult to precisely identify the cause of the different adaptation time in kinematics, as many parameters could be involved.

In contrast to other works [31, 34, 35], the soleus steady-state activity was not reached by all the subjects within two powered sessions in [33], despite the controller and the protocol being the same as the ones in [31]. A difference between the two studies is the number of PAMs used to provide the powered push-off in the PAFO (two parallel PAMs in [33] and a single one in [31]). In their work, Kao et al. [33] pointed out that the amount of assistive torque provided by the PAFO could have influenced the time needed by the subjects to reach the steady state. However, this hypothesis is not confirmed by the results reported in Table 5.

Sawicki et al. [36] found that the subjects in their work reached a steady state, in the metabolic cost of walking, in about 90 min (divided into three testing days in which the subjects walked with the powered PAFO for 30 min). The authors suggested that the subjects could have needed a relatively long adaptation time because they walked with bilateral PAFOs. However, more recent studies [40, 41, 43] seem to contradict this hypothesis.

### The effects of the push-off actuation timing

One of the most decisive variables affecting the results of powered walking is the actuation timing [18, 21, 40]. The efficacy of the assistance provided at the ankle highly depends on the synchronization between the actuation of the PAFO and the user’s motion. An inaccurate timing impedes the user in his/her movements and interferes with the action of the biological muscles. When walking with an augmentation PAFO, the push-off onset timing has a considerable impact on the user [18]. The effects of different onset timings on the metabolic cost and muscle activation of the users are discussed in the sequel. However, it is important to notice that the studies analyzed in this subsection utilize different actuation setups, i.e. actuators design and control strategy, as highlighted in Table 6 and Table 7. The differences in the mechanical design and the control architecture of different actuators greatly affect their behavior and, by extension, their effect on the user’s effort. For this reason, the comparisons presented in this subsection are given with the aim of providing an overview of the similarities and the divergences between the results obtained in different studies. Nevertheless, the reader is warned to read the analysis with caution.

**Table 6:** Comparison of the effects of different actuation timings on the user’s effort during level walking

Ref.	Protocol	Onset timings	Peak timings	Offset timings	Metabolic cost	Soleus activity
					w.r.t. U	w.r.t. U
Galle, 2017 [21] ♢	4 onset conditions, fixed offset	**Earliest (36%)**	/	64%	-(14% - 18%)	**-40% (peak) in Earliest**, smaller reduction for later onset timings
		**Early (42%)**			**-(16% - 21%)**	
		Late (48%)			-(16% - 17%)	
		Latest (54%)			-8%	
Malcolm, 2013 [18] ♢	5 onset conditions, fixed offset	13%	/	63%	-5%	/
		23%			-12%	
		34%			-15%	
		**43%**			**-17%**	
		54%			-2%	
Zhang, 2017 [46] □	Iterative learning to find optimal onset, peak, offset timings to reduce metabolic cost	**17% - 37%** (varied among subjects)	48% - 55% (varied among subjects)	59% - 65% (varied among subjects)	**-(14% - 37%)** (varied among subjects)	/
	or soleus activation	**9%**	44%	61%	/	**-41% (rms)**

The effects on the metabolic cost and soleus activity of healthy users during powered walking are reported with respect to the unpowered condition (U). The onset, peak and offset timings are expressed as a percentage of the gait time. In each study, the onset timings in bold are the values found to be the optimal ones to minimize the metabolic cost or the soleus activation in the subjects during walking

Symbols (♢,□) indicate studies performed by the same research group on similar actuation setups. Symbols are consistent between tables. In the works in which different experiments were performed (for example, different onset timings) the common information between the experiments (for example, same offset timing) is reported only once

**Table 7:** Comparison of the effects of different actuation timings on the user’s effort during uphill walking

Ref.	Incline	Protocol	Onset	Peak	Offset	Metabolic	Soleus
			timings	timings	timings	cost w.r.t. U	activity w.r.t. U
Galle, 2015 [19] ♢	15%	4 onset conditions, fixed offset	19%	/	66% - 67%	-11%	/
			26%			-12%	/
			**34%**			**-12%**	similar
			41%			-10%	/
Zhang, 2017 [46] □	10%	Iterative learning to find optimal onset, peak, offset timings to reduce metabolic cost	**42%**	55%	65%	**-26%**	/

The effects on the metabolic cost and soleus activity of healthy users during powered uphill walking are reported with respect to the unpowered condition (U). The onset, peak, and offset timings are expressed as a percentage of the gait time. The onset timings in bold are the values found to be optimal to minimize the metabolic cost of walking in the works assessing multiple onset conditions

Symbols (♢,□) indicate studies performed by the same research group on similar actuation setups. Symbols are consistent between tables. In the work in which different experiments were performed (for example, different onset timings) the common information between the experiments (for example, same offset timing) is reported only once

#### Effects of Onset Timing in Level Walking

Table 6 collects the results of the studies assessing the effects of the actuation timing on the user’s metabolic cost of walking and soleus activity. From the reported results in the table, two considerations can be drawn:

- The optimal onset timing to minimize the metabolic cost of walking is not consistent between studies. In their work, Zhang et al. [46] determined a range of optimal onset timings which minimized the metabolic cost of walking on different subjects. In opposition to their result, the optimal onset timing found by Malcolm et al. [18] and Galle et al. [21], in their works, does not fit in this range.
- Zhang et al. [46] found that the optimized onset timing to reduce the soleus activity is earlier than that with optimal results for metabolic cost reduction in the same study. The limitation of this experiment is that only one subject participated in it; however, a similar trend for bigger reductions in soleus activation with earlier onset timings, can be noticed in the results reported by Galle et al. [21].

As previously mentioned, it is important to highlight that while the actuation platform used in [18] and [21] is similar, it differs from the one used in [46]. As a consequence, the differences in the optimized onset timing between these studies could be partially explained by the use of different setups.

Galle et al. [21] also assessed the effects of different onset timings on the activation of other lower limb muscles. The gastrocnemius tended to have more reduced activation for later (54%) onset timings, while the reduction of the tibialis anterior and biceps femoris activation appeared bigger for onset timings between 42% and 48% of the gait cycle. No significant correlations were found between onset timing and muscle activation for the vastus lateralis, rectus femoris, and gluteus maximus.

#### Effects of onset timing on uphill walking

Few studies have assessed the effects of the different onset timing on users walking uphill (Table 7). Looking at the resulting optimized onset timings in these works and comparing them to the ones found by the same research groups for level walking, one can notice a disagreement in the findings. While Zhang et al. [46] found that a later onset timing was more beneficial for uphill walking as compared to level walking, Galle et al. [19] found the opposite result. However, the inconsistent results found by the two studies could be due to several factors. As for level walking, the fact that the two studies used different actuation setups could have had an influence on the outcomes. Furthermore, only one subject participated in the test performed by Zhang et al. [46]. In addition to this, the different inclinations tested in the two studies could be a cause for the differences in the results. For these reasons, it is very difficult to draw conclusions from the results reported in Table 7 on the influence of the onset timing on the metabolic cost of uphill walking.

### The effects of push-off assistance magnitude

Another parameter that alters the effect of the PAFO on users is the assistance magnitude. The influence of this parameter has been analyzed in few studies in unloaded [20, 21] and loaded [23, 25] walking conditions. The outcomes of these works are compared with the results obtained by other studies in Table 8 and Table 9, respectively and discussed in this section. However, it is important to note that the differences in the actuation setups, i.e. mechanical design and control, of the PAFOs used in different studies could have had an influence on the reduction of the user’s effort while walking.

**Table 8:** Comparison of the effects of different assistance magnitude on the user’s effort during level walking

Ref.	Assistance magnitude		Metabolic cost w.r.t. U	Soleus activation w.r.t. U
Galle, 2017 [21] ♢	double pos.	0.21 W/kg	-(14%-18%)	reduced with increased power level (peak)
		0.41 W/kg	-(16%-21%)	
		0.50 W/kg	-(16%-17%)	
Jackson, 2015 [20] □	net	-0.05 W/kg	+5%	-9% (rms)
		0 W/kg	0%	-14%
		0.09 W/kg	-9%	-32%
		0.18 W/kg	-17%	-36%
		0.25 W/kg	-17%	-45%
Mooney, 2014 [48] ⋈	pos.	0.15 W/kg	-14%	/
Mooney, 2016 [49] ⋈	pos.	0.1 W/kg	-14%	/
Sawicki, 2008 [36] △	pos.	0.24 W/kg	-15%	-18% (rms)
Sawicki, 2009 [37] △	pos.	0.17-0.23 W/kg	-(8%-12%)	-(11%-20%) (rms)
Sawicki, 2009 [38] △	pos.	0.23 W/kg	-13%	-25% (rms)

The assistance magnitude is defined as the average power provided by the PAFO per stride. The effects on the metabolic cost and soleus activity of powered walking are reported with respect to the unpowered condition (U). The results of the study by Sawicki et al. [38] are reported only for level walking. Jackson et al. [20] studied the effects of net assistance magnitude, however, for positive levels the negative average power is negligible, thus, their results can be compared to the ones of the other studies. Galle et al. [21] assessed the effects of the double positive assistance magnitude, i.e. the sum of the assistance magnitude for the two legs. Regarding the results of the soleus activation, the table reports whether the peak or the rms values are considered in the different studies

Symbols (♢,□,⋈,△) indicate studies performed by the same research group on similar actuation setups. Symbols are consistent between tables. In the works in which different experiments were performed (for example, different levels of assistance magnitude) the common information between the experiments (for example, same type of assistance magnitude) is reported only once

**Table 9:** Comparison of the effects of different assistance magnitude on the user’s effort during loaded walking

Ref.	Load	Assistance magnitude		Metabolic cost w.r.t. U	Metabolic cost w.r.t. N
Lee, 2016 [23] ⋆	23 kg	double neg	10% of double pos	-11%	/
	(29% bodyweight)		20% of double pos	-11%	
			30% of double pos	-15%	
		double pos	same value for all neg conditions		
Malcolm, 2017 [25] ⋆	23 kg	double neg	-0.015 W/kg	-11%	/
	(29% bodyweight)		-0.016 W/kg	-12%	
			-0.027 W/kg	-11%	
			-0.037 W/kg	-15%	
		double pos	0.19 W/kg		
Mooney, 2014 [47] ⋈	23 kg	double pos	0.27 W/kg	/	-8%
	(27% bodyweight)				
		double neg	0 W/kg		

The assistance magnitude is defined as the average power provided by the PAFO per stride. The effects on the metabolic cost of powered walking are reported with respect to the unpowered (U) or normal walking (N) condition. It should be noted that the amount of load as a percentage of the subjects’ bodyweight is comparable between different studies

Symbols (⋆,⋈) indicate studies performed by the same research group on similar actuation setups. Symbols are consistent between tables. In the works in which different experiments were performed (for example, different levels of assistance magnitude) the common information between the experiments (for example, same load) is reported only once

Galle et al. [21] assessed the effects of the double positive assistance magnitude, i.e. the average positive power provided by the PAFO per stride, summed up for the two legs. Jackson et al. [20] assessed the effects of the net assistance magnitude, i.e. the average net power per stride per leg. In the positive net assistance magnitude levels in [20], the amount of negative power was very small compared to the positive one, thus, the results found in the two works can be compared.

The results obtained in [20, 21] show that a bigger reduction of the subjects’ soleus activation during powered unloaded walking, is obtained with a bigger positive assistance magnitude. In opposition to this, it seems that a medium level of assistance magnitude leads to a bigger reduction in the metabolic cost of walking as compared to lower and higher levels of assistance magnitude. However, the results found by Sawicki et al. in different studies [36–38] seem to contradict this outcome (Table 8); the works with higher assistance magnitude showed a bigger reduction in the metabolic cost of powered walking.

Providing positive assistance magnitude to the ankle joint of a user can help in reducing the metabolic cost of loaded walking also, as shown in Table 9 by the results of Mooney et al. [47]. Recently, some studies were performed to assess the effects of negative assistance magnitude on the ankle joint during loaded walking [23, 25] (Table 9). Contrary to what was tested by Jackson et al. [20] during unloaded walking, in these studies the negative assistance magnitude was provided in association with the positive assistance magnitude. However, to highlight the effects of the negative assistance magnitude on the user’s effort, in both studies, the positive assistance magnitude level was kept constant throughout different negative assistance magnitude conditions. In both cases, a trend of bigger reduction in the metabolic cost of walking for higher levels of negative assistance magnitude was found, however, the differences between conditions were not significant. Nevertheless, these results indicate the possibility of achieving a bigger reduction in metabolic cost by combining positive and negative assistance magnitude at the user’s ankle joint.

### The type of controller influences the human response

As previously introduced, the proportional myoelectric controller (PMc) and the phase-based controller (P-Bc) are the main types of controllers used in PAFOs. The benefits and drawbacks of the two controllers have already been discussed in several studies [40–43] and they are summarized below:

- The PMc has the advantage of being better synchronized with the user, resulting in a more physiological controller because the user has direct control over the timing and amplitude of the actuation;
- The P-Bc has lower complexity and it does not need sensors on the user’s limbs, since they can, in general, all be placed on the device.

The determination of the specific effects of the two controllers on the user is a very interesting point since it could help define whether one of the two controllers is more suitable for a specific goal. A discussion about this topic is provided in the sequel.

#### Ease of adaptation to the controller

A highly debated point is one that refers to the ease of adaptation of the subjects to the two controllers. On one hand, the PMc is considered to be more natural for subjects, making it easier for them to learn how to walk with the device [71]. On the other hand, the benefit of the P-Bc is that the assistance provided by the PAFO is constant throughout the experiment and independent of the user’s response, making it is easier to apply [43]. There are a few studies that have tried to compare the influence of the type of controller on the adaptation time.

Cain et al. [35] found no significant differences between the adaptation time of the subjects walking with a PAFO controlled by a PMc and a P-Bc (Table 5). However, the different implementation of the two controllers within this study could have affected the results, thus, complicating the comparison of the specific effect of the controllers.

A more systematic comparison of an adaptive gain PMc (Ag-PMc) and a P-Bc was performed by Koller et al. [41, 42]. The two controllers in these studies were designed to have the same average actuation signal. However, the authors did not report information regarding the adaptation time for the two controllers.

#### Influence of the type of controller on the user’s effort

Koller et al. [41, 42] assessed the influence of different controllers on the metabolic cost of walking and lower limb muscle activation of users (Table 2). The two controllers seem to be equivalent in terms of the reduction of the metabolic cost of walking, while different results are found on the effects of the controllers on muscle activation [41, 42].

The bigger reduction of the user’s muscle activation found by Koller et al. [41, 42] with the P-Bc, led the authors to conclude that a PMc would be preferable for rehabilitation purposes, to prevent the patient from being passively driven by the PAFO. Furthermore, it could be helpful in rehabilitation procedures that are based on error augmentation techniques [28]. On the other hand, a P-Bc would be a more favorable choice in patients with altered lower limb muscular activity [43]. In this case, the disrupted pattern of the patient’s muscular activity could lead to inappropriate timing of the assistance provided by a PMc-driven PAFO to the user. In addition to this, due to the lower complexity of its implementation, the P-Bc can be a more suitable solution for devices providing assistance in daily life activities.

### Quantification of the metabolic advantage of the PAFO

In the studies performed by Mooney et al. [47–49], the Augmentation Factor (AF) was introduced as a metric to predict the metabolic impact of walking with an exoskeleton in contrast to normal walking. The AF is calculated as shown in Eq. 1:

1 $$ AF = \frac{p^{+} + p^{dis}}{\eta} - \sum_{i=1}^{4}\beta_{i}m_{i}   $$

where *p*^+^ and *p*^*dis*^ are the average positive and negative mechanical power provided by the exoskeleton, per leg in a stride, i.e. the positive and negative assistance magnitude, *η* is human muscle-tendon efficiency, *m*_*i*_ and *β*_*i*_ are the added mass and the location factor related to the *i*^*th*^ human segment, respectively. The muscle-tendon efficiency *η* is found to be equal to 0.41 in [18, 30, 37]; the location factors *β*_*i*_ are equal to 14.8 W/kg, 5.6 W/kg, 5.6 W/kg and 3.3 W/kg for the foot, shank, thigh and waist, respectively [69]. The relationship between the AF and the metabolic advantage *MetAdv*_*norm*_ of powered walking was determined in [48] and given in Eq. 2:

2 $$ MetAdv_{norm} = 1.1 AF - 5   $$

in which the positive values of *MetAdv*_*norm*_ represent a decrease in the metabolic cost of powered walking as compared to normal walking.

Galle et al. [21] determined another formula to calculate the metabolic advantage of powered walking with respect to the unpowered condition (*MetAdv*_*unpow*_), as described in Eq. 3:

3 $$\begin{array}{*{20}l} \begin{aligned} \small MetAdv_{unpow} =& -\left(0.0088 + 9.1 \cdot P^{+} + 5 \cdot P^{+2} +\right.\\& \left.- 0.64 \cdot Ton \cdot P^{+} \,+\, 0.0077 \cdot Ton^{2} \cdot P^{+}\right) \end{aligned}  \end{array} $$

In contrast to the formulation by Mooney et al. (Eq. 1 and 2), Eq. 3 relates the metabolic advantage of the PAFO to both the onset timing (*Ton*) and the positive assistance magnitude summed for the two legs (*P*^+^). Furthermore, the weight of the device is not considered, since it affects both the unpowered and powered conditions.

## Assistive and rehabilitation PAFOs

The possible advantages of using a PAFO as a tool to assist or rehabilitate subjects with ankle deficiencies are well illustrated in literature [68, 70, 72]. However, only few studies have been performed till date that test the capabilities of PAFOs on these subjects (Table 3 and 4).

Contrary to augmentation PAFOs, the main goal of assistive and rehabilitation PAFOs is to improve the altered gait pattern of users with weakened ankle capabilities. Although there is no agreement on what the metrics are for assessing the improvement, Ward et al. [60] designated a list of performance metrics to assess the capabilities of a PAFO on stroke patients while walking. However, other studies performed on impaired patients assessed only a few parameters that are included in this list. For this reason, only the most common criteria to assess the performance of assistive and rehabilitation PAFOs on elderly and impaired patients will be discussed in the sequel. These parameters are the ankle range of motion (RoM), maximum plantarflexion angle, occurrence of drop foot, walking speed, gait symmetry, and step cadence. In addition to this, the effects of powered walking on a user’s effort, i.e. muscle activation and metabolic cost of walking, will be presented and compared to the results obtained on healthy subjects. However, only few studies have assessed these parameters.

### Effects on spatio-temporal parameters

The main goal of assistive and rehabilitation PAFOs is to improve the altered gait pattern of their users, by correcting the ankle RoM and preventing the occurrence of drop foot in subjects with weakened ankle dorsiflexors. Another goal of these PAFOs is to improve the subjects’ walking speed, which is usually reduced by the impairment [68]. Some subjects with weakened ankle capabilities present an asymmetry in the gait pattern between the left and the right leg. In these cases, the assistance provided by the PAFO aims to reduce this asymmetry.

In the following section, the effects of assistive and rehabilitation PAFOs on these parameters are discussed.

#### Walking pattern improvement with assistive PAFOs

The results reported in Table 10 show that an assistive PAFO can be used to prevent the occurrence of drop foot, while not hindering the ankle joint in plantarflexion [51, 54]. Furthermore, Blaya et al. [51] and Sawicki et al. [65] showed that the improvements in ankle kinematics can also be achieved at faster walking speeds than the user’s preferred one with respect to the cases in which an elastic cord [65] or a conventional AFO [51] were used to assist drop foot (Table 10).

**Table 10:** Effects of powered walking on the ankle kinematics and gait pattern of impaired patients

Ref.	Ankle RoM	Drop foot occurrence	Asymmetry
Awad, 2017 [63] (Str)	*↑* Df in swing (4.78 deg) w.r.t. U	/	/
Awad, 2017 [64] (Str), tethered	*↑* Df in swing (5.33 deg) w.r.t. U	/	*↓* in peak propulsion w.r.t. U (20%)
	*↑* peak propulsion (11%) w.r.t. U		*↓* in propulsion impulse w.r.t. U (19%)
Awad, 2017 [64] (Str), untethered	*↑* Df in swing (4.9 deg) w.r.t. U	/	*↓* in propulsion w.r.t. U (16.3%)
	*↑* peak propulsion (13%) w.r.t. U		
	*↑* propulsion impulse (14%) w.r.t. U		
Bae, 2015 [26] (Str)	/	/	*↓* in step time by 6% w.r.t. N
			*↓* in stance time by 4% w.r.t. N
			*↓* in propulsion by 7% w.r.t. N
Bae, 2018 [27] (Str)	/	/	*↓* in positive body CoM power by 39% w.r.t. U
			*↓* in ankle power by 40% w.r.t. U
Blaya, 2004 [51] (Dfi)	*↑* Df in swing (37%-200%) for all speeds w.r.t. A; *↑* Pf in stance (25%-89%) for all speeds w.r.t. A	eliminated in P and A at slow and self-selected speed; at fast speed *↓* by 67% in P w.r.t. A	*↓* in step length w.r.t. A and N (up to 100%); *↓* in step time w.r.t. A (up to 75%) and N (up to 94%)
Sawicki, 2006 [65] (iSCI)	*↑* PO angle w.r.t. U (up to 14.5 deg) and N (up to 10.7 deg) for all speeds in therapist-control, results in patient-control lower than in therapist-control;	/	/
	better improvements at lower speeds		
Shorter, 2011 [52] (Pfi)	kinematics minimally affected by PAFO (slightly *↓* Df)	/	/
Shorter, 2011 [53] (Pfi)	*↓* Df	/	shifted from 2% longer right step in N to 2% and 6% longer left step in U and P
Shorter, 2011 [53] (Dfi)	corrected RoM during swing; *↓* RoM in late stance	reduced	shifted from 1% longer right step in N to 4% and 2% longer left step in U and P; eliminated for step time
Takahashi, 2015 [39] (Str)	RoM more dorsiflexed in U and P w.r.t. N	/	/
Yeung, 2017 [54] (Str)	no improvements in PO	reduced	/

The ankle kinematics, occurrence of drop foot, and gait symmetry are compared between the following conditions: walking with powered assistive PAFOs (P), unpowered condition (U), walking with a conventional AFO (A), or walking without devices (N)

Dfi, Pfi: Dorsiflexion-/plantarflexion-impaired patients; iSCI: Incomplete spinal cord injury patients; Str: Stroke patients; Df, Pf: Dorsiflexion and plantarflexion; PO: Push-off; CoM: Center of mass

Different effects of the PAFO on the gait symmetry of the user have been found by different studies [51, 53]. When walking with the assistive PAFO, the subjects in [51] experienced a highly reduced asymmetry in step length and step time between the affected and unaffected leg, with respect to normal and conventional AFO conditions. A more modest improvement in the step and stance time asymmetry was showed by the users participating in the study presented in [26]. In [53], only one of the two subjects participating in the experiment showed a slight asymmetry in the step time, which was eliminated during powered walking. In contrast, both subjects increased their asymmetry in step length by augmenting the step length on the leg without the PAFO.

Another interesting result was reported by Awad et al. [64]. In their work, the authors assessed the effects of two different onset timings on the propulsion symmetry of stroke patients. The results obtained in this study greatly varied with different patients. More specifically, only one of the subjects participating in the test benefited equally from both onset timings. Some patients benefited from both onset timings, but the earlier one was more beneficial than the later one. However, other patients benefited from the later onset timing, while the earlier onset timing worsened the propulsion symmetry.

#### Walking pattern improvement with rehabilitation PAFOs

Table 11 shows the effect of some weeks of training with a PAFO, on the ankle kinematics and gait pattern of impaired users. In general, all the reported studies found an improvement in the walking conditions of the patients when comparing walking cadence and speed, step length asymmetry, and ankle joint kinematics after the weeks of training with the powered PAFO.

**Table 11:** Effects on ankle kinematics and gait pattern of impaired patients after training with a PAFO

Ref.	PAFO training	Comparison	Cadence	Walking speed	Asymmetry	Ankle RoM
Kim, 2007 [59] (Hem)	4 weeks	pre-/post- N U and P walking	*↑* by 4% in P, similar in N	*↑* by 35% in P *↑* by 27% in N	in step length: *↓* by 45% in P and 85% in N	/
Kim, 2011 [58] (Hem)	4 weeks	pre-/post- N U and P walking	*↑* by 5% in P and by 2% in N	*↑* by 38% in P *↑* by 27% in N	in step length: *↓* by 28% in P and 80% in N	/
Ward, 2007 [62] (Str)	8 weeks	6 min walk: pre-/post- N	/	10 months training pre-PAFO: increased by 225% (plateau reached); 2 months training PAFO increased by extra 48% (linear increase)	/	Better kinematics with PAFO than without, even if circumduction because of bulkiness PAFO
		3 meters walk: pre-/post- N		results in line with training pre-PAFO		
		timed up and go: pre-/post- N		results in line with training pre-PAFO		
Ward, 2011 [60] (Str)	3 weeks	pre-/post- P walking	*↑* by 6%-13%, (depending on the subject)	/	/	*↑* RoM by 32%-338% (depending on the subject)

The effects on walking cadence, walking speed, and asymmetry between the two legs are reported. The duration of the training with the rehabilitation PAFO is given

Hem: Hemiplegic patients; Str: Stroke patients; P, U, N: Powered PAFO, unpowered PAFO and no devices condition. In the work in which different experiments were performed (different comparisons) the common information between the experiments (for example, same PAFO training) is reported only once

Interesting results are reported by Kim et al. [58, 59], who showed that the walking performance of hemiplegic subjects was improved between the first and fourth week of the training, both for the powered and the normal walking conditions. Another outcome worth pointing out is presented by Ward et al. [62]. In their study, the authors compared the results of two months of training with the PAFO with the ones obtained in the previous 10 months of training without the PAFO on a stroke patient. During the training without the PAFO, the subject seemed to have reached a plateau in terms of the increase of walking speed. However, in the two successive months, the subject’s walking speed increased further, despite the intensity and the duration of training with the PAFO being similar to the one without the device.

### Effects on a user’s effort

Only a few studies that have been performed with assistive PAFOs assessed the effect of a powered push-off during level walking on muscle effort and the metabolic cost of walking of elderly [55, 56] and impaired subjects [27, 39, 63, 65] (Table 12). Although reducing the user’s effort is not the main goal of assistive PAFOs, assessing the effect of powered walking on the muscle activity of impaired users is of great importance. This is because the active involvement of the impaired subjects is fundamental in locomotor training [72].

**Table 12:** Comparison of the effects of powered walking on the efforts of elderly and impaired users

Ref.	Subjects	Pos. assistance magnitude	Onset time	Metabolic cost		Soleus activation	
				w.r.t. N	w.r.t. U	w.r.t. N	w.r.t. U
Awad, 2017 [64] ⋆	Str	/	28% or 37%	/	-10%	/	/
Bae, 2018 [27] ⋆	Str	/	individualized onset timings from [64], varied between 26% and 40%	/	-10%	/	/
Galle, 2017 [21] ♢	H	0.21 W/kg	48%	lower but not significant	-16%	lower but not significant	lower but not significant
Galle, 2017 [56] ♢	E	0.11 W/kg	49%	lower but not significant	-12%	/	/
Norris, 2007 [55] △^∗^	H	0.059 W/kg	/	+8%	-5%	/	/
	E	0.043 W/kg	/	higher but not significant	lower but not significant	/	/
Sawicki, 2006 [65] △	iSCI	/	34% (therapist)	/	/	similar	-(7%-19%)
		/	44% (patient)	/	/	similar	-(12%-27%)
Takahashi, 2015 [39] ≀	Str	0.018 - 0.023 W/kg	32%	higher but not significant	lower but not significant	-35%	-24%

The metabolic cost and the soleus activity during the powered condition are compared to the ones during the unpowered (U) and normal walking (N) conditions. The results of the studies with healthy young subjects performed in [55] and [21] are added as a comparison to the studies on elderly subjects since they have similar assistance magnitudes and onset timings. Contrary to the other studies, the positive assistance magnitude in [21] is given as the sum of the positive assistance magnitudes of the two legs

E: Ederly subjects; iSCI, Str: Incomplete spinal cord and stroke patients; H: Healthy young subjects. Symbols (⋆,♢,△,≀) indicate studies performed by the same research group on similar actuation setups. △^∗^ indicate a design based on △, but not coming from the same research group. Symbols are consistent between tables. In the works in which different experiments were performed (for example, different onset timings) the common information between the experiments (for example, same type of subjects) is reported only once

#### Muscle activation

Sawicki et al. [65] and Takahashi et al. [39] assessed the effect of walking with powered plantarflexion on the lower limb muscle activation of patients with incomplete spinal cord injuries and strokes, respectively (Table 12). The proportional myoelectric controlled PAFO in [39] reduced the activation of the paretic soleus of the user as compared to normal walking. On the contrary, Sawicki et al. [65] did not report a difference in the muscle activation during powered walking as compared to normal walking with a PAFO controlled by a push-button controller. The controller in [65] could be associated to a phase-based controller, since the authors reported that the PAFO actuation was consistent during the experiment. Furthermore, in both controllers, the assistance of the PAFO is independent of the user’s effort. Thus, the reported results seem to contradict what was anticipated from the experiments on healthy users, regarding the effects of the type of controller on users’ muscle activation. However, neither of the studies assessed the achievement of the steady state in muscle activation in the subjects. Subjects in [39] walked with the PAFO only for five minutes, per condition. Supposing that impaired patients need the same amount of adaptation time as healthy subjects (Table 5), it is probable that they did not reach the steady state in muscle activity in this time. Thus, the soleus activity of these subjects could have kept reducing until the steady state was reached. The subjects in [65] underwent longer sessions, thus, the reported results could be closer to the steady-state ones.

#### Metabolic cost of walking

The effect on the metabolic cost of a powered push-off in assistive PAFOs was assessed by Galle et al. [56] and Norris et al. [55] on elderly subjects and by Takahashi et al. [39], Awad et al. [64] and Bae et al. [27] on stroke patients (Table 12). Table 12 compares the effect of powered walking on elderly and young subjects. A similar effect is obtained in the two groups, but the latter achieve a bigger reduction in metabolic cost with comparable assistance parameters. In [56] and [55] the authors suggested that elderly subjects could need a longer period of time to adapt to powered walking, however, none of the studies assessed the achievement of a steady state in these subjects.

Takahashi et al. [39] found no differences between the powered and unpowered conditions, in the metabolic cost of walking of stroke patients. As seen in healthy subjects, the metabolic cost of walking of these patients tended to decrease with multiple sessions, although the differences were not statistically significant. As discussed above, the powered trials in [39] lasted only five minutes, which could have been insufficient to lead the subjects to a steady state in terms of metabolic cost. Thus, it is possible that different results could have been achieved if the subjects had walked longer.

In opposition to this, Awad et al. [64] and Bae et al. [27] found a reduction in the metabolic cost of powered walking as compared to unpowered walking, in stroke patients who walked eight minutes per day with the powered PAFO. The reductions in metabolic cost reported in [27, 64] are given for the most beneficial onset timing for each user.

## Discussion

Some common trends of the effects of PAFOs on healthy and weakened users have been identified in the previous sections. In addition to them, some divergences can be noticed in the results presented by different studies. A discussion about these findings is addressed in this section.

### The importance of assessing user adaptation to the PAFO

As previously introduced, the assessment of the adaptation of the user to powered assistance is important to compare the effects of the PAFO in different studies. This is due to the changes in the kinematics, muscle activation, and metabolic cost of walking between the adaptation and the steady-state period [18, 31–36, 40, 43].

Some differences have been noted in the time to achieve a steady state in works with similar protocols (Table 5). This suggests that the adaptation time could be influenced by some assistance parameters. However, the details regarding the assistance parameters are not always reported in the studies. Thus, it is complicated to compare their effects on the adaptation time.

The time needed to achieve a steady state during powered walking has not been assessed in elderly and impaired users. Assessing whether the subjects achieved a steady state is important in assistive and rehabilitation PAFOs to evaluate their effects. For example, assessing the achievement of a steady state at the end of each training session with rehabilitation PAFOs could help distinguish whether the changes in the gait pattern of the subject between sessions are an effect of the robotic training or of the adaptation of the user to the PAFO.

### The influence of the assistance parameters on the metabolic advantage of the PAFO

As already discussed in the previous sections, it is not easy to define a trend for the influence of the onset timing and the assistance magnitude on the reduction of the soleus activation and the metabolic cost of walking. One of the reasons for this is the fact that the comparison between studies performed on different actuation setups, is not always straightforward.

As presented above, two formulae that relate the metabolic advantage of the PAFO to the assistance parameters have been proposed by Mooney et al. [47–49] (Eqs. 1 and 2) and Galle et al. [21] (Eq. 3), respectively. Looking at the formulae proposed by the different authors, one can notice that they are in disagreement regarding the effect of the positive assistance magnitude on the metabolic advantage of the PAFO. In Eqs. 1 and 2, a higher positive assistance magnitude would result in a bigger metabolic advantage of the PAFO on the user, while the formulation in Eq. 3, for a fixed onset timing, is a quadratic function of the positive assistance magnitude. As already reported, an inconsistency in the effect of higher levels of assistance magnitude can be noted between the results reported by Sawicki et al. [36–38] and Galle et al. [21]. A difference between these studies is in the onset timing, which is around 14% in [36–38], while it ranges between 36% and 48% in [21]. The results by Mooney et al. [49] also do not match with Eq. 3. In this study, a very early onset timing is used (the PAFO starts providing plantarflexion torque, even if slightly, at heel strike), which according to Eq. 3 should lead to an increase of the metabolic cost of walking, but the subjects experienced a reduction of the metabolic cost of walking as compared to the unpowered condition. This could suggest an interplay in the influence of the onset timing and the positive assistance magnitude on the metabolic advantage of the PAFO, for which the effect of the assistance magnitude is different for earlier onset timings in contrast to later timings. The investigation of the impact of the assistance magnitude on earlier onset timings could also explain the earlier range of optimized onset timings found by Zhang et al. [46]. Another noticeable fact is that the formula presented by Galle et al. [21] includes only the positive assistance magnitude in the calculation of the metabolic advantage of the PAFO, but, as presented by Lee et al. [23] and Malcolm et al. [25], the negative assistance magnitude can also play a role in the reduction of the metabolic cost of walking. However, all these studies were performed on different actuation setups. As already explained, this fact amplifies the uncertainties in terms of comparing their results; thus, complicating the determination of a general equation to describe the metabolic advantage of a PAFO. All these considerations suggest that a single assistance parameter cannot be considered as independent when analyzing the effects of a PAFO on the user and underline the fact that the comparison of these effects between different studies has to be made carefully.

### Lack of studies on weakened users

The results reported in the previous section highlight the advantages of using a PAFO to improve the ankle kinematics and walking speed of its users and to rehabilitate impaired subjects. However, while numerous studies have analyzed the effects of the assistance parameters on the biological effort of healthy young users [18–21, 35, 41, 42, 46, 56], there is a lack of research carried out on elderly and impaired users. Although the effects of a PAFO on the metabolic cost of walking of elderly and impaired subjects resemble the ones obtained on healthy subjects, some differences can be noted. The different response of elderly subjects with respect to young users to the plantarflexion assistance provided by the PAFO (Table 12), highlights that it is not possible to extrapolate the influence of powered assistance on these users from the results obtained on healthy young subjects. As already explained, this difference could be due to longer time requirements of the elderly to adapt to powered assistance. Another explanation could be that the assistance parameters have a different effect on the metabolic cost of walking in the two groups of subjects. This hypothesis implies that the formula of the metabolic advantage should be revised for different groups of subjects. Although assistive and rehabilitation PAFOs do not aim to reduce the metabolic cost of walking [28], the determination of this formula would be a useful tool for their design, to give an idea of the necessary assistance to be provided to compensate for the metabolic energy spent by the user to carry the added mass of the PAFO.

### Impact of walking with a powered PAFO on more proximal joints

**Fig. 2:**
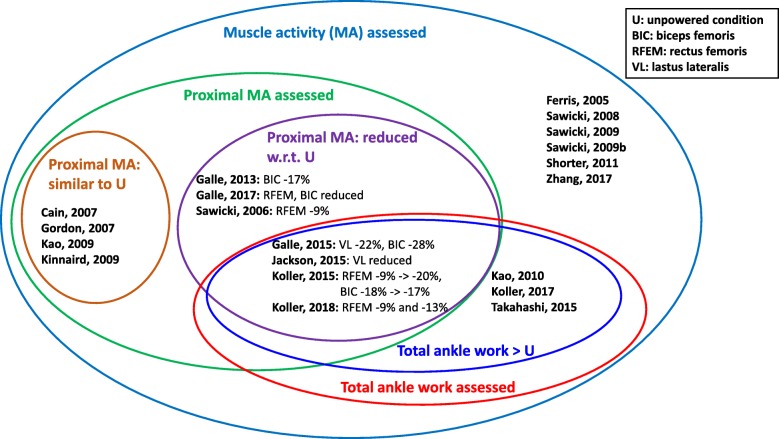
Visualization of the studies assessing the muscle activity of the lower limbs muscles. Some of the works assessed only the activity of the muscles related to the ankle joint [29, 36–38, 46, 52], while other works also assessed the activity of more proximal muscles [19–21, 31, 32, 34, 35, 40, 42, 43, 65]. Between these studies, all the works measuring the total (biological plus exoskeletal) ankle work [19, 20, 33, 39–42] found that the total work was increased during the powered condition with respect to the unpowered one

Some of the presented studies on PAFOs assess the activity of lower limb muscles that are not directly related to ankle movements, such as the vastus lateralis, vastus medialis, biceps femoris, rectus femoris, medial hamstring, lateral hamstring, and gluteus maximus [19–21, 31, 32, 34, 35, 40, 42, 43, 65]. Figure 2 shows a schematic of the results of these works. Some studies found that, after the adaptation period, the activity of the proximal muscles during powered walking was similar to unpowered walking [31, 32, 34, 35], but others reported a different result [19–21, 40, 42, 43, 65].

Galle et al. [19] did not observe a reduction of the soleus activation with respect to unpowered walking when the subjects walked uphill with the powered PAFO providing push-off assistance, but they noticed a reduced activity of the vastus lateralis and biceps femoris. The authors hypothesized that this effect was due to a proximal mechanism that used the additional action at the ankle joint to redirect the center of mass in the single support phase reducing the effort in the hip joint, which is heavily loaded during uphill walking, instead of the ankle joint. A similar effect was found also by Koller et al. [40]; the subjects, walking with the PAFO providing assistive push-off, initially decreased the activation of the soleus by 20% with respect to unpowered walking, but with further powered sessions, the activity of the soleus increased until it was only 11% lower than during unpowered walking. Furthermore, the increase in the soleus muscle activation was accompanied by an increasing reduction of the activation of the rectus femoris (from 9% to 20%) and the biceps femoris (17% - 18%) as well as by a reduced average hip positive power with respect to unpowered walking. The same authors, in a different work [42], reported that subjects walking with powered PAFOs reduced the muscular activity of both the soleus and the rectus femoris with respect to unpowered walking. Jackson et al. [20] noticed that an increase of the average net power provided by the PAFO during powered push-off walking resulted in a decrease of the activity of the vastus lateralis. As in [19], the authors associated this effect to the proximal action of the PAFO in redirecting the center of mass. A reduction of the hip positive power when walking with the powered PAFO providing push-off was also found by Mooney et al. [49], but in this study, the muscle activity of the subjects was not recorded.

Some of the works that reported a reduced activity of more proximal muscles also measured the total (biological plus exoskeletal) ankle work during powered and unpowered walking. A common finding of these works is that the subjects’ total ankle work during powered walking was higher than it was during unpowered walking. This effect would suggest that in these studies, the enhancement of human capabilities was achieved by the PAFO augmenting the action of the human ankle, instead of replacing part of it. Other works found an increase in the total ankle work during powered walking with a PAFO [33, 39, 41], however, in these studies, the activation of more proximal muscles was not assessed.

From these results, it seems that there are two possible effects of augmentation PAFOs providing powered push-off to the user: the PAFO could replace part of the biological ankle work, or it could augment it and assist not only the ankle joint, but also the hip joint. However, due to the small sample of works that analyzed the effects of the PAFOs on more proximal joints, it is difficult to explain which are the elements that make the PAFO augment rather than replace the biological ankle work.

### Importance of assessing human-robot interaction

A very important aspect that should be studied in PAFOs is the interaction of the device with the user’s body. This interaction can be divided into two levels: a physical and a neural one. The physical interaction plays a very important role in accomplishing the successful transmission of torques and forces from the device to the user [73]. It is known that the interfaces, i.e. the connections between the user and the device, act as a series compliance which can dissipate as much as 50% of the mechanical power from the device [74, 75]. However, few studies analyze the human–robot interaction during experiments performed on users.

An example of the importance of assessing the physical human–robot interaction when analyzing the effects of the PAFO on the user is given by Van Dijk et al. [50]. In their study, they found that the metabolic cost of two of the three healthy users walking with a PAFO, was increased during the powered condition as compared to the unpowered one (Table 2). One of the possible reasons that explains this result is the deviation found between the ankle angle of the PAFO and of the user during the powered condition. As explained by the authors, these deviations could have been caused by deformations in the PAFO structure and in the user’s soft tissues, which could have dissipated part of the energy transmitted from the device to the user and, thus, prevented the reduction of the metabolic cost of walking.

Another important aspect that should be considered in the development of PAFOs is how the nervous system will respond to the provided assistance, i.e. the neural interaction. Kao et al. [32] showed that the assistance provided by a PAFO does not always result in a reduction of the muscle activation of the muscle working in synergy with the assistance (Table 1). In their test, two groups of healthy users were assisted in dorsiflexion. One group received the assistance only during swing, while the other group was assisted both during swing and the loading response. In the powered condition, the activation of the tibialis anterior during the loading response in the second group was reduced; however, in both groups, its activity remained similar to the unpowered condition, during swing. The authors explained the unaltered activity of the tibialis anterior during swing as a result of the fact that the increased total (biological plus PAFO) action at dorsiflexion produces an exaggerated dorsiflexion, which does not cause any harm to the subject. On the other hand, an exaggerated dorsiflexion during the loading response would have caused instability to the subject; for this reason, the tibialis anterior activation was reduced in this phase when the assistance was provided.

A similar consideration was made by Kinnaird et al. [34]. In their study, they provided plantarflexion assistance to healthy users during walking with a PMc-driven PAFO in which the assistance was proportional to the activity of the medial gastrocnemius. The result of this study showed that the users reduced the activity of the soleus, i.e. the muscle working in synergy with the mechanical output of the PAFO, more than the medial gastrocnemius, i.e. the muscle used to control the PAFO (Table 1). This result showed that the primary focus of the nervous system of the users was to alleviate the increased plantarflexion. However, the reduction in the activity of the medial gastrocnemius also showed that the nervous system could learn the relationship between this muscle and the assistance provided.

## Future directions

PAFOs have been shown to have great potential to enhance the capabilities of healthy users and assist or rehabilitate the ankle joints of weakened ones. However, more research is necessary to improve the understanding of the impact of these devices on the user.

From the results presented in this paper, it seems that the adaptation time of healthy users is influenced by the assistance parameters. The determination of a relationship between these variables is complicated by the lack of information regarding the onset timing and assistance magnitude in most of the studies. More research should be performed to determine the influence of the assistance parameters on the adaptation time, both on healthy and weakened users.

Future studies should be conducted with more combinations of onset timing and assistance magnitude to assess their interplay in the determination of the metabolic advantage of the PAFO.

More studies are needed on elderly and impaired subjects. The small number of studies performed on these subjects makes it difficult to accurately compare the results obtained by different studies. These studies should focus on the influence of the assistance parameters on the effect of the PAFO on the user. Furthermore, the time needed by these subjects to reach a steady state during powered walking should be assessed.

An interesting topic to be investigated is also the influence of the type of controller on the response of weakened subjects to the assistance provided by the PAFO. The determination of distinctive effects of the different controllers would define whether a specific controller is more suitable for a certain group of subjects or for a precise objective.

Another open question to be addressed is how long the subjects can retain the steady-state walking pattern that has been learned during powered assistance. This would be particularly interesting for rehabilitation PAFOs since it could determine the frequency of robotic rehabilitation sessions.

Additionally, the effect of powered walking on more proximal joints should be studied to explain the parameters determining whether a PAFO will augment or replace the biological ankle work.

Furthermore, another aspect that should be better studied is the physical interaction of the device with the user, which is of great importance in the understanding of the effect of a PAFO on the user.

## Conclusion

The performance of PAFOs in terms of healthy and weakened users varies between studies with similar protocols and goals. The effect of powered walking on users is influenced by a set of key factors, which have been identified in this article. It has been shown that these factors mutually impact the performance of PAFOs on users, thus, the influence of each one of them cannot be considered independently from the others. In this paper, it has been highlighted that the comparison of the results of different studies is not always straightforward. This is due to the fact that the behavior of a PAFO is greatly influenced by the dynamics of its specific actuation setup and the comparison of the results obtained from different actuation setups is difficult to make. This comparison would be facilitated with the development of a standard methodology to benchmark actuators, which is, however, still an open issue [76, 77]. From the comparison of the outcomes of different studies, it can be seen that the effects of a PAFO on weakened subjects cannot be extrapolated from the ones obtained on healthy ones.

The results presented in this paper lead to the conclusion that more experiments need to be performed on elderly and impaired subjects. In the future, studies should specify the parameters used in the protocol (type of controller, onset timing, and assistance magnitude) and report, together with the results, whether the subjects had reached a steady state in the experiment. This is particularly relevant for studies performed on elderly and impaired users. Assessing the influence of these parameters on these users would simplify the analysis of the effects of powered walking.

## Abbreviations

Ag-PMC: Adaptive gain proportional myoelectric controller
BPnA: Bidirectional rotary pneumatic actuator
CoM: Center of mass
PAFO: Powered ankle-foot orthosis
PAM: Pneumatic artificial muscle
PMC: Proportional myoelectric controller
PnA: Pneumatic actuator
P-Bc: Phase-based controller
P-btnc: Push-button controller
RoM: Range of motion
SEA: Series elastic actuator
SOM: Spring over muscle actuator
StA: Stiff actuator
